# Supplementary material for: KSHV activates unfolded protein response sensors but suppresses downstream transcriptional responses to support lytic replication
Source: PLoS Pathog. 2019 Dec 2;15(12):e1008185. doi: 10.1371/journal.ppat.1008185 (PMC6907875; doi:10.1371/journal.ppat.1008185)
Supplement: Attachment — Submitted filename: Johnston UPR response to reviewers June 27 2019.docx [file ppat.1008185.s001.docx]

**Response to Reviewers detailing the revisions for #PPATHOGENS-D-18-01985**

**“KSHV activates unfolded protein response sensors but suppresses downstream transcriptional responses to support lytic replication”**

Dear Editor,

Thank you for considering our manuscript at *PLoS Pathogens.* We also thank the reviewers for their thorough reviews and constructive suggestions that have had the effect of strengthening our manuscript. We performed and included several entirely new experiments and modified the manuscript to the best of our ability to accommodate all three reviewers. Overall, we think that the manuscript has been significantly improved and hope it meets the expectation for publication in *PLoS Pathogens*.

Briefly, the most significant improvements to the manuscript address the primary concern of **all 3 Reviewers** with regard to mechanistic understanding of viral UPR modulation. We now show that:

1) UPR transcriptional suppression is **independent of SOX,** the KSHV host shutoff protein **(New multipanel Figure 5)**. **Reviewer 3 encouraged us to consider the effects of host shutoff in our model.** During KSHV lytic replication the viral host shutoff endonuclease SOX targets the majority of host mRNAs for degradation, although significant minority of host mRNAs are ‘escapees’ that are not degraded. SOX-mediated bulk mRNA degradation indirectly causes accumulation of host RNA-binding proteins in the nucleus and reduces the recruitment of RNA polymerase II to host promoters, causing global repression of host transcription. Remarkably, we found that ectopically-expressed SOX could not inhibit basal, thapsigargin-induced, or tunicamycin-induced UPR gene expression in our system (either compared to mock-treated cells or cells expressing an RNA endonuclease mutant version of SOX (P176S)). This SOX construct was functional, and able to reduce levels of canonical SOX substrate mRNA GAPDH. These findings indicate that SOX expression has little impact on UPR signaling irrespective of the mode of ER stress induction, and is unlikely to play a significant role in inhibiting UPR transcriptional responses during lytic replication.
2) We also firmed up the data on KSHV inhibition of the ISR in direct response to **Reviewer 1**. We now demonstrate that despite strong PERK activation and eIF2α phosphorylation during the lytic cycle, **ATF4 protein** but not its mRNA, and **mRNA levels of the ATF4 target gene CHOP**, are greatly diminished **(new Figure 1B).**
3) Finally, in response to **Reviewer 2**, we tested the ability of ectopic XBP1s to inhibit KSHV virion production in TREx-BCBL1-RTA cells. Previously, we showed that expressing XBP1s via a dox-inducible or constitutive CMV promoter in iSLK.219 cells inhibited virion production at a late stage of replication, in a dose-dependent manner. Surprisingly, XBP1s did not block virion production in TREx-BCBL1-RTA cells at all. We conclude that XBP1s exerts cell-type specific inhibition of KSHV virion production, even though it can nicely reactivate KSHV from latency in both cell types. We do not yet understand the nature of this selective XBP1s-mediated inhibition of KSHV replication in the iSLK.219 cells, but the diminished accumulation of structural proteins ORF26 and ORF65, and the altered electrophoretic mobility of ORF65 and the tegument protein ORF45, have focused our attention on potential post-translational modifications and turnover of viral structural proteins as a starting place for future studies (none of these alterations were observed in the TREx-BCBL1-RTA cell model upon ectopic XBP1s expression). We thank Reviewer 2 for their helpful suggestion.

Please find below our response to reviewers’ comments, outlined point-by-point as requested. We have embedded several of the new figures in this letter to facilitate your review:

***Reviewer #1:***

*It is unclear why Tg-induced activation of RTA (which triggers viral reactivation) does not induce expression of even early genes such as ORF45. Along these lines, if UPR stress alone can induce lytic reactivation then why is there a need to co-treat with Dox? It would seem like a more straightforward approach to track UPR outputs under conditions of Tg-induced reactivation.  In this context, RNAseq type experiments could be done to get a more comprehensive picture of UPR-linked transcriptional changes +/- KHSV reactivation.*

We specifically chose the dox-inducible iSLK.219 and TREx-BCBL1-RTA cell models to study how viral replication affects the UPR and ISR. By inducing with dox, we could efficiently reactivate from latency without using any of the common chemical stimuli (NaB, VPA, TPA/ionomycin) that have broad and dramatic effects on stress signaling and host cell gene expression. Indeed, thapsigargin treatment would cause lytic reactivation, but it would also cause so much stress that it would likely be impossible to discern potential viral perturbations in UPR/ISR. Instead, we elected to use thapsigargin throughout the study as a positive control to induce UPR and ISR activation, and to reveal how the virus could be undermining the responsiveness of these pathways at different stages of lytic replication. Thapsigargin was typically delivered in short pulses for 2 or 4 h prior to harvesting cell lysates for RNA or protein analysis. A brief 2 h pulse with thapsigargin in the absence of dox, as shown in Fig. 1A, is not long enough to allow significant accumulation of ORF45 or other KSHV lytic proteins. Later in the study, we devised multiple strategies to promote lytic reactivation via ectopic XBP1s expression (Figures 8-10), once again avoiding the broad impacts on host cell signaling and gene expression that would result from poisoning the SERCA pump with thapsigargin. In sum, these studies were specifically designed to avoid the broad and cytotoxic effects of thapsigargin and other chemical stimuli of lytic reactivation.

*In figures 2 and 3 there appears to be translational attention upon Tg and DOX treatment of the cells, suggesting that eIF2α phosphorylation might indeed be occurring. Although this is not easily visible in the westerns, P- eIF2α is notoriously difficult to observe. The authors may consider complementing their western analyses with S35 methionine plus labeling to better visualize potential translational shutoff. Additionally, more analytic methods using eIF2α alpha screens have been used to measure phosphorylation Sidrauski et al. Elife 2013. Downstream targets of ATF4 (i.e. CHOP) should be examined to confirm that there no functional ATF4 protein being made.*

We have consistently observed the accumulation of phosphorylated eIF2α during early lytic replication, which is sustained throughout later stages, both in the iSLK.219 and TREx-BCBL1-RTA cell models. Examples of this can be found in Figs. 1A, 2, and 3. We observed diminished Tg-induced ATF4 protein during lytic in this same set of Figures, suggesting that KSHV may be breaking the ISR downstream of the eIF2α phosphorylation step, and preventing uORF scanning required to make ATF4. To strengthen this point, we have performed **new experiments presented in Figure 1B** that demonstrate that at 24 h post-reactivation in the TREx-BCBL1-RTA cells, thapsigargin treatment elicits ~4-fold less CHOP transcription than in mock-treated cells. Tg-induced ATF4 mRNA levels are also significantly reduced during the lytic cycle compared to controls, although this effect is less dramatic than the reduction in CHOP.

*Several of the blots in figure 3 are poor quality and hard to interpret, especially the blots for PERK and ATF6. The authors may consider using a reporter gene assay as published in Gallagher et al. Elife 2016 to confirm no activation in the context of reactivation. This would help explain the increase in XBP1u as mentioned in text.*

We agree that these PERK and endogenous ATF6 western blots are difficult to interpret, and we have improved clarity by removing them from this multipanel figure, narrowing the focus to the IRE1/XBP1s pathway, which is the main point of this figure anyway. We note that available antibodies for phosphorylated forms of PERK and IRE1 and total ATF6 protein are quite poor. This is a challenge that the whole field faces, and is the underlying reason why we rely on total PERK and IRE1 blots to reveal phosphorylation-dependent electrophoretic mobility shifts in these proteins.

*Over expression of XBP1s caused a dramatic reduction of viral titers during reactivation. Chronic over expression of XBP1s has been shown to induce pro-apoptotic pathways e.g. the JNK pathway. The authors should examine cellular viability during overexpression to rule out toxicity issues that might lead to reduced viral titers. This should also be done for the drug treatment or knockdown of ATF6, PERK and IRE1. Furthermore, it would be worthwhile to look at other UPR branches during overexpression to evaluate their contributions to this phenotype.*

We were initially concerned as well about potential cytotoxic effects of ectopic XBP1s expression, but we have not observed any overt signs of cytotoxicity in the iSLK.219 cell models, beyond the virus-induced cytotoxicity observed in late stages of lytic replication, which XBP1s expression does not alter. Our subsequent experiments in the TREx-BCBL1-RTA cell model (**new Figure 10)** reveal that ectopic XBP1s expression did not affect virion production in these cells, further strengthening our conclusion that XBP1s expression is not inherently cytotoxic in these cell models.

*Please number western blot lanes when mentioning specific lanes in the text, i.e. for Figure 2.*

We have revised the images to include lane numbers to aid interpretation.

***Reviewer #2:***

*A number of the experiments looking at virion production were done in various engineered SLK cells. The SLK cells tend to be relatively tightly latent, so they are not the best system to test activation of KSHV by XBP-1 (as in Figure 8), especially as XBP-1s has been shown to induce KSHV RTA in other systems.  Ideally this should be looked at in other backbone cell lines; at a minimum, this limitation should be acknowledged.*

We thank the reviewer for this excellent suggestion, which prompted us to test the ability of ectopic XBP1s to inhibit KSHV virion production in TREx-BCBL1-RTA cells **(see new Figure 10, above).** Previously, we showed that expressing XBP1s via a dox-inducible or constitutive CMV promoter in iSLK.219 cells inhibited virion production at a late stage of replication, in a dose-dependent manner. Surprisingly, XBP1s did not block virion production in TREx-BCBL1-RTA cells at all. We conclude that XBP1s exerts cell-type specific inhibition of KSHV virion production, even though it can nicely reactivate KSHV from latency in both cell types. We do not yet understand the nature of this selective XBP1s-mediated inhibition of KSHV replication in the iSLK.219 cells, but the diminished accumulation of structural proteins ORF26 and ORF65, and the altered electrophoretic mobility of ORF65 and the tegument protein ORF45, have focused our attention on potential post-translational modifications and turnover of viral structural proteins as a starting place for future studies. For reference, please see a modified version of Figure 9C here that is less tightly cropped to better display the alterations in protein species detected by the ORF65 antibody. Clearly something interesting is happening to ORF65 in the context of XBP1s overexpression, with bulk accumulation of a ~28 kDa band, seemingly at the expense of higher MW bands (marked with *) at ~32 kDa, ~34 kDa and ~58 kDa that normally accumulate in cells reactivated by ectopic RTA expression. None of these alterations were evident in the TREx-BCBL1-RTA cell model upon ectopic XBP1s expression. We thank Reviewer 2 for their helpful suggestion.

*The authors inhibit PERK with GSK2606414 (line 352).  However, this drug may have off-target effects; this should be acknowledged.*

Reviewer #2 correctly points out that the PERK inhibitor drug can have off-target effects, although the 500 nM dose that we use is in line with other studies in the literature that have shown selective inhibition. Cells were also treated with ISRIB in parallel in this experiment, which suppresses the ISR downstream of PERK-mediated eIF2α phosphorylation by promoting stability of the decameric eIF2B GEF and recharging of the ternary complex. Thus, the similar inhibition of virion production by PERKi and ISRIB provide complementary information about KSHV reliance on PERK activity for efficient lytic replication.

*The Discussion rambles some, especially when a number of speculations regarding other viruses are discussed.  It should be tightened.*

We thank Reviewer 2 for this constructive advice. We have truncated the discussion significantly to improve readability. Forgive us, we sometimes get carried away…..

*One of the lytic proteins produced during KSHV lytic replication is shut-off exonuclease (SOX).  It would seem possible that the function of SOX is playing a role in mediating the failure of UFR effectors during lytic replication.  The authors should consider this possibility.*

We have performed several new experiments (**new Figure 5, described below in detail**) which demonstrate that SOX has no effect on UPR transcription in several different experimental setups. We conclude that these effects are likely SOX-independent. Our current focus is on identifying and characterizing viral UPR regulating gene products.

***Reviewer #3:***

*In the results section for Figure 1, they state that “Tg-treated lytic cells also displayed a reduced total IRE1a and XBP levels event though XBP1 mRNA splicing was similar to TG-treated latent cell”. What is the role of host shut off induced by KSHV in this effect.  This could simply be due to host shut-off not specific effects on the UPR downstream response*.

We thank the reviewer for this excellent suggestion, which prompted the experiments displayed in the **new Figure 5**. During KSHV lytic replication the viral host shutoff endonuclease SOX targets the majority of host mRNAs for degradation, although significant minority of host mRNAs are ‘escapees’ that are not degraded. SOX-mediated bulk mRNA degradation indirectly causes accumulation of host RNA-binding proteins in the nucleus and reduces the recruitment of RNA polymerase II to host promoters, causing global repression of host transcription. Remarkably, we found that ectopically-expressed SOX could not inhibit basal, thapsigargin-induced, or tunicamycin-induced UPR gene expression in our system (either compared to mock-treated cells or cells expressing an RNA endonuclease mutant version of SOX (P176S)). This SOX construct was functional, and able to reduce levels of canonical SOX substrate mRNA GAPDH. These findings indicate that SOX expression has little impact on UPR signaling irrespective of the mode of ER stress induction, and is unlikely to play a significant role in inhibiting UPR transcriptional responses during lytic replication.

*In Figure 1A the high molecular weight band reacting with the antibody for ATF6 does not appear in the HA tagged version making it more likely that it is a lytic induced cross-reactive band not a form of ATF6 and not likely relevant.*

We agree that this is possibly a cross-reactive species and not relevant. We have modified the Figure 1A annotation and legend to clarify this.

*Figure 1C again raises the question of what is the potential role of host shut-off by KSHV on the levels of these host RNAs?*

We have addressed the role of host shutoff by studying SOX, as described above and in the new Figure 5. SOX is unable to inhibit the accumulation of UPR responsive gene products in several assays. We conclude that UPR suppression must be a function of other viral gene products. The identification and characterization of these viral UPR regulators is the focus of our ongoing studies.

*In Figure 2, the effect of the perk inhibitor on eif2alpha phosphorylation is minimal without Thapsigargin.  Quantitation of the effect would be helpful to determine the magnitude and significance of the effect.*

We agree. The effects of the selective PERK inhibitor drug on PERK phosphorylation is dramatic across the board, but effects on eIF2α phosphorylation are modest in the absence of thapsigargin. At the same time, it is clear that eIF2α phosphorylation is a feature of lytic replication that begins early. Although we have clearly observed that PERK activity contributes to eIF2α phosphorylation during the lytic cycle, we believe that the residual eIF2α phosphorylation observed during PERK inhibition is the result of another eIF2α kinase. One good candidate is GCN2, which can be activated in response to DNA damage; such damage is a feature of KSHV infection, as described in the following 2 papers:

[Activation of **DNA Damage** Response Pathways during Lytic Replication of **KSHV**.](https://www.ncbi.nlm.nih.gov/pubmed/26057167)

Hollingworth R, Skalka GL, Stewart GS, Hislop AD, Blackbourn DJ, Grand RJ.

Viruses. 2015 Jun 5;7(6):2908-27. doi: 10.3390/v7062752.

PMID: 26057167

[Localization of Double-Strand Break Repair Proteins to Viral Replication Compartments following Lytic Reactivation of Kaposi's Sarcoma-Associated Herpesvirus.](https://www.ncbi.nlm.nih.gov/pubmed/28855246)

Hollingworth R, Horniblow RD, Forrest C, Stewart GS, Grand RJ.

J Virol. 2017 Oct 27;91(22). pii: e00930-17. doi: 10.1128/JVI.00930-17. Print 2017 Nov 15.

PMID: 28855246

*For figure 2 they state that “treating lytic cells with PERKi revealed that even though ATF4 fails to accumulate during lytic replicaton with or without Tg treatment, PERK-mediated phosphorylation of eIF2alpha may still inhibit bulk protein synthesis as evidenced by the increased levels of the early viral gene product ORF45”.  Showing one viral protein does not in any way provide evidence of this statement.  A minor point is that they also reference figure 2A here and there is only one panel for figure 2.*

We have revised the text to clarify our description of this experiment. The new statement reads as follows: “*Furthermore, lytic cells treated with PERKi in the presence or absence of Tg also showed increased levels of ORF45 (Fig. 2, compare lanes 9 vs 10 & lanes 11 vs 12), which suggests that even though ATF4 fails to accumulate, PERK-mediated phosphorylation of eIF2α may still impede viral protein synthesis to some extent*.”

*In figure 3 they state that maximal XBP1s splicing occurred at 1 hour for Tg but by eye it looks like it is 4 hours.*

We thank the reviewer for their careful analysis of this semiquantitative XBP1 mRNA splicing assay. We have revised the text to indicate that the majority of XBP1s splicing occurred at 1 h (rather than maximal).

*The PERK and phosphor PERK blot in figure 3 is uninterpretable as it is too light to determine changes in protein levels.*

All of the labs in this field are challenged by poor available antibodies. However, Reviewer #3 is right that these PERK blots are so faint that they defy interpretation. We have deleted them.

*Figure 5:  Are the effects of inhibition of the three different response proteins additive or is this all the same point of inhibition?  This could be an important point.*

It is standard practice in the field to genetically-silence or inhibit one UPR sensor at a time in cells, rather than all three. We believe that silencing all UPR sensors would be quite toxic to the cell, and we are not aware of any studies to date in which this has been reported. There is an important point to be made about compensatory effects following inhibition of one arm of the UPR; these pathways are tightly interconnected.
